# The protease activated receptor 2 - CCAAT/enhancer-binding protein beta - SerpinB3 axis inhibition as a novel strategy for the treatment of non-alcoholic steatohepatitis

**DOI:** 10.1016/j.molmet.2024.101889

**Published:** 2024-02-01

**Authors:** Villano Gianmarco, Novo Erica, Turato Cristian, Quarta Santina, Ruvoletto Mariagrazia, Biasiolo Alessandra, Protopapa Francesca, Chinellato Monica, Martini Andrea, Trevellin Elisabetta, Granzotto Marnie, Cannito Stefania, Cendron Laura, De Siervi Silvia, Guido Maria, Parola Maurizio, Roberto Vettor, Pontisso Patrizia

**Affiliations:** 1 Dept. of Surgical, Oncological and Gastroenterological Sciences, University of Padova, Italy; 2 Dept. of Clinical and Biological Sciences, University of Torino, Italy; 3 Dept. of Molecular Medicine, University of Pavia, Italy; 4 Dept. of Medicine, University of Padova, Italy; 5 Dept. of Biology, University of Padova, Italy

**Keywords:** Serpins, Genetically manipulated mice, Experimental NASH, Transcription factors, Therapeutic drugs

## Abstract

**Objective:**

The serine protease inhibitor SerpinB3 has been described as critical mediator of liver fibrosis and it has been recently proposed as an additional hepatokine involved in NASH development and insulin resistance. Protease Activated Receptor 2 has been identified as a novel regulator of hepatic metabolism. A targeted therapeutic strategy for NASH has been investigated, using 1-Piperidine Propionic Acid (1-PPA), since this compound has been recently proposed as both Protease Activated Receptor 2 and SerpinB3 inhibitor.

**Methods:**

The effect of SerpinB3 on inflammation and fibrosis genes was assessed in human macrophage and stellate cell lines. Transgenic mice, either overexpressing SerpinB3 or carrying Serpinb3 deletion and their relative wild type strains, were used in experimental NASH models. Subgroups of SerpinB3 transgenic mice and their controls were also injected with 1-PPA to assess the efficacy of this compound in NASH inhibition.

**Results:**

1-PPA did not present significant cell and organ toxicity and was able to inhibit SerpinB3 and PAR2 in a dose-dependent manner. This effect was associated to a parallel reduction of the synthesis of the molecules induced by endogenous SerpinB3 or by its paracrine effects both *in vitro* and *in vivo*, leading to inhibition of lipid accumulation, inflammation and fibrosis in experimental NASH. At mechanistic level, the antiprotease activity of SerpinB3 was found essential for PAR2 activation, determining upregulation of the CCAAT Enhancer Binding Protein beta (C/EBP-β), another pivotal regulator of metabolism, inflammation and fibrosis, which in turn determined SerpinB3 synthesis.

**Conclusions:**

1-PPA treatment was able to inhibit the PAR2 - C/EBP-β - SerpinB3 axis and to protect from NASH development and progression, supporting the potential use of a similar approach for a targeted therapy of NASH.

## Highlights

• SerpinB3 is a mediator of liver inflammation, steatosis and fibrosis.
• The Protease Activated Receptor 2 (PAR2) is activated by SerpinB3.
• The CCAAT Enhancer Binding Protein-beta is essential for SerpinB3 synthesis.
• 1-Piperidine Propionic Acid inhibits PAR2 and protects from non-alcoholic steatohepatitis.

## Graphical abstract

**Figure:**
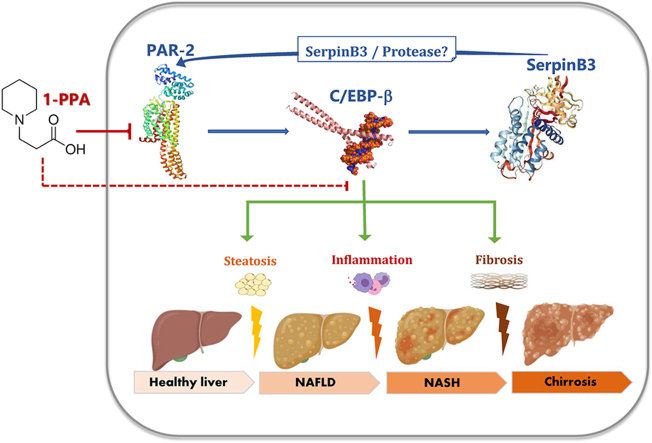

## Abbreviations

Serpins: serine-protease inhibitors
TIMP-1: tissue inhibitor of metalloproteases type 1
MCD: methionine- and choline-deficient
NAFLD: non-alcoholic fatty liver disease
MASLD: metabolic dysfunction-associated steatotic liver disease
NASH: non-alcoholic steatohepatitis
TGF: transforming growth factor
α-SMA: α-smooth muscle actin
HIF: hypoxia-inducible factor
1-PPA: 1-Piperidine Propionic Acid
C/EBP-β: CCAAT Enhancer Binding Protein beta
KO: knock-out
WT: wild-type
CDAA: choline-deficient aminoacid-defined
SAT: subcutaneous adipose tissue
VAT: visceral adipose tissue
BAT: brown adipose tissue
WAT: white adipose tissue
LAP: Liver Activating Protein
LIP: Liver Inhibitory Protein
qRT-PCR: quantitative real-time PCR reaction

## 1 Introduction

The liver has a key role in maintaining metabolic homeostasis, particularly in controlling and modulating lipid and glucose metabolism, as well as in drug detoxification. In addition, the liver has also unique immunological aspects that include fetal hematopoiesis, induction of immune tolerance but, at the same time, also a very efficient innate immunity, to mention just a few [1]. Chronic liver injury, irrespective of etiology, can lead to the induction of persistent inflammatory response and dysregulated activation of wound-healing repair, with hepatic macrophages and myofibroblasts cooperating in a finely regulated manner and playing a prominent role in sustaining fibrogenic progression of the disease [[1], [2], [3], [4], [5]]. Interactions among different hepatic cell populations and release of a large number of mediators (i.e., growth factors, chemokines, pro-inflammatory cytokines, plasma proteins, reactive oxygen species, hepatokines, adipokines and more) as well as the involvement of multiple molecular pathways and of genetic and environmental factors are believed to play a major role in chronic liver disease progression [[2], [3], [4], [5]].

SerpinB3 is a peculiar member of the family of **ser**ine-**p**rotease **in**hibitor**s** (Serpins) which is almost undetectable in normal murine and human liver [6]. However, under conditions of chronic liver injury of different etiology, SerpinB3 expression is significantly upregulated in hepatocytes in human liver biopsies [7,8] and even in liver carcinogenesis [[9], [10], [11]]. More recently, SerpinB3 has been proposed as a critical mediator of liver inflammation and fibrosis. Regarding this aspect, SerpinB3 up-regulates the expression of transforming growth factor (TGF)-β1 in chronic liver disease [7] and exerts a direct pro-fibrogenic action on human liver myofibroblasts in culture by strongly up-regulating the expression of pro-fibrogenic genes (including collagen type 1A1, α-smooth muscle actin or α-SMA, TGF-β1, and tissue inhibitor of metalloproteases type 1 or TIMP-1) [12]. The pro-fibrogenic action of SerpinB3 was mechanistically confirmed using transgenic mice overexpressing SerpinB3 in hepatocytes that were submitted to two distinct experimental protocols of chronic liver injury, including the protocol of methionine- and choline-deficient (MCD) diet able to induce fatty liver and steatohepatitis [12]. In addition, studies on transgenic mice, either overexpressing SerpinB3 or carrying Serpinb3 deletion, and *in vitro* studies on human macrophage cell lines have shown that SerpinB3 can operate as a pro-inflammatory mediator in two models of progressive non-alcoholic fatty liver disease (NAFLD) [13], a definition that has been recently replaced by the new definition of “*metabolic dysfunction-associated steatotic liver disease*” (MASLD) [14]. The involvement of SerpinB3 in NAFLD and in its progressive form non-alcoholic steatohepatitis (NASH) is relevant since NAFLD is emerging as the major cause of chronic liver disease worldwide, with epidemiological data indicating a 30 % prevalence in the general population and an even higher prevalence (>70 %) in obese individuals and Type II diabetes patients, which represent the typical NAFLD/NASH patients [5,15]. Moreover, despite promising indications from preclinical studies, at present no pharmacological therapy has been approved for NASH treatment [16,17]. NAFLD and its progression towards NASH is a resultant of a complex scenario involving excess energy intake, insulin resistance and inflammation, resulting in an increased flux of fatty acids to the liver, dysregulation of hepatic lipid metabolism and de novo lipogenesis [18].

SerpinB3 expression is up-regulated by hypoxic conditions and its increased transcription is mediated by specific binding of hypoxia-inducible factor (HIF)-2α to the SerpinB3 promoter [19]. Moreover, SerpinB3 has been shown to act as a paracrine mediator able to affect the behavior of surrounding cells by differentially up-regulating, in normoxic conditions, HIF-1α and HIF-2α [20]. In particular, SerpinB3 through HIF-1α up-regulation can favor cell survival in a harsh (i.e., hypoxic) microenvironment by inducing early cellular metabolic switch to glycolytic phenotype [20]. By contrast, SerpinB3 can induce HIF-2α stabilization through direct NEDDylation and this has been suggested to promote proliferation of liver cancer and to favor hepatocellular carcinoma progression [20]. Considering a putative role in relation to NAFLD/NASH patients, which are mostly obese individuals and/or type II diabetes patients, by inducing HIF-2α stabilization SerpinB3 may represent a factor able to deeply affect lipid metabolism. Indeed, studies employing mouse models with Cre-lox mediated deletion of VHL, HIF1α and/or HIF2α have shown that HIF-2α, rather than HIF-1α, plays a major role in regulating hepatocellular lipid accumulation by various mechanisms, including up-regulation of lipid biosynthetic pathways and suppression of fatty acid oxidation [21,22]. Moreover, studies in transgenic mice carrying hepatocyte-specific deletion of HIF-2α and analysis performed on NAFLD/NASH patients have shown that HIF-2α activation is a key feature of both experimental and human NASH [23] and is also involved in NASH-related carcinogenesis, where HIF-2α levels were found to be strictly associated with hepatocyte production of SerpinB3 [24]. Due to the interconnections between SerpinB3, HIF-2α-mediated regulation of lipid metabolism and NAFLD/NASH progression, we proposed in a recent study that SerpinB3, which is mainly expressed and released by stressed/injured hepatocytes, may represent an additional novel hepatokine [13]. Hepatokines are defined as proteins synthetized and secreted by hepatocytes, able to affect metabolic processes through autocrine, paracrine and endocrine signaling and know to play a key role in orchestrating whole-body energy metabolism and to be involved in NASH and insulin resistance [[25], [26], [27]].

To further investigate the role of SerpinB3 in NASH progression, in the present study we have investigated a novel therapeutic strategy for the treatment of NASH using 1-Piperidine Propionic Acid (1-PPA), a small molecule patented and proposed as SerpinB3 inhibitor [28,29]. More recently, 1-PPA was also found to sterically inhibit the protease activated receptor 2 (PAR2) [30], a cell surface sensor of extracellular inflammatory and coagulation proteases, that has been also recognized as a new regulator of hepatic metabolism [31,32]. PAR2 indeed not only controls cholesterol homeostasis and lipid metabolism [31], but also suppresses glucose internalization, glycogen storage, and insulin signaling [32]. In the present study we have assessed whether 1-PPA may be able to inhibit steatosis, inflammation and fibrosis, the hallmarks of progressive NASH, *in vitro* and after parenteral administration in transgenic and wild type mice with diet-induced NASH. The use of Serpinb3 knock-out mice and of 1-PPA allowed us to confirm the essential role of the antiprotease activity of this serpin in the pathogenesis of NASH. Moreover, by investigating the efficacy of 1-PPA in inhibiting NASH progression, we also found that 1-PPA, through the inactivation of PAR2, determines a down-regulation of the early transcription factor CCAAT Enhancer Binding Protein beta (C/EBP-β). This transcription factor is highly expressed not only in adipose tissue, but also in liver, kidney, intestine, pancreatic islets, and innate immune cells. It regulates the expression of several genes involved in development, immune function, regeneration, differentiation, and metabolism [33].

Since both PAR2 and C/EBP-β are activated in proinflammatory conditions [34,35] and recent data also support their possible relevance in the process of fibrogenesis [36,37], our results provide evidence for the pivotal role of SerpinB3 in the activation of the PAR2 - C/EBP-β axis and for its effective inhibition by the small molecule 1-PPA.

## 2 Results

### 2.1 SerpinB3 and development of murine NASH

**Figure 1:**
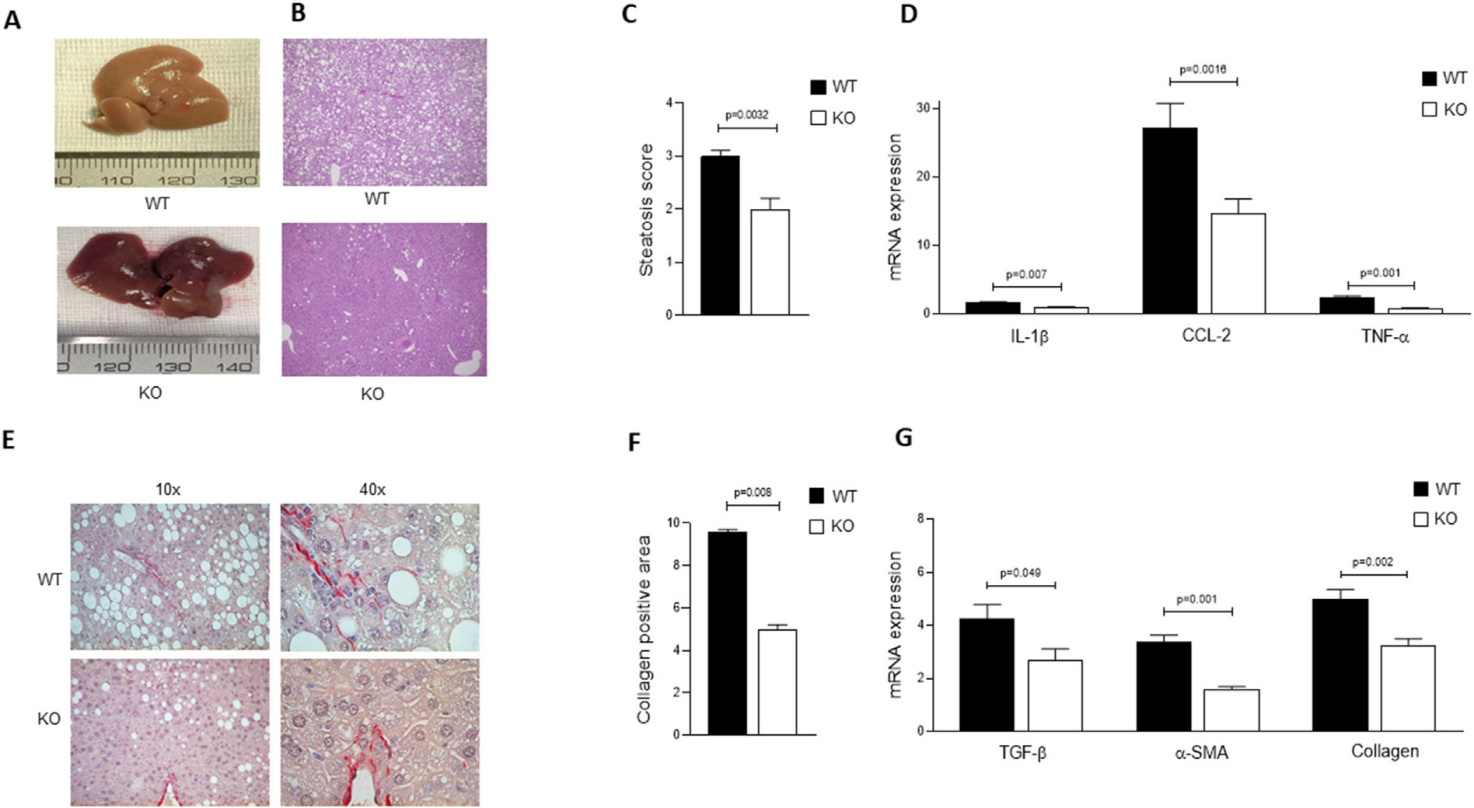
**SerpinB3, lipid accumulation and development of murine NASH.** Representative examples of morphological findings at sacrifice of the liver of a BALB/c wild-type mouse (WT) and of a mouse deficient of the reactive site loop of Serpinb3a (KO), fed with CDAA diet. A) liver macroscopic features, B) liver histology after hematoxylin eosin staining, E) collagen deposition assessed by Sirius red staining. The pictures provide evidence of the lower extent of steatosis in KO mouse, confirmed by the lower levels of steatosis scores detected by liver histology in KO mice (7 animals) compared to WT mice (7 animals) (C). Similar findings are reported for the profile of inflammatory genes (D), including IL-1β, CCL2 and TNF-α, and of fibrosis genes (G), including TGF−β, α-SMA and collagen 1A1. Densitometric analysis of collagen deposition measured after Sirius red staining (F) shows the lower extent of collagen deposition detected in KO mice, compared to WT mice. The results are reported as mean ± SEM (Unpaired t test with Welch's correction). Levels of mRNA gene expression are reported as 2^- ΔΔCT^. (For interpretation of the references to color in this figure legend, the reader is referred to the Web version of this article.)

To mechanistically investigate the role of SerpinB3 in relation to the development of murine NASH, we took advantage of mice genetically manipulated to carry a deletion in the reactive site loop of Serpinb3a [38], the closest isoform to human SerpinB3, hereafter indicated as knock-out (KO) mice, as well as their related wild-type (WT) mice on the common genetic background BALB/c. These KO mice, recently used to characterize the pro-inflammatory role of Serpinb3 in murine NASH [13], were fed for this part of the study on choline-deficient aminoacid-defined (CDAA) dietary protocol to induce experimental NASH, very effective and reliable to induce steatosis, inflammatory response and fibrosis [12,13,23]. The lack of the antiprotease activity of Serpinb3a in KO vs WT mice, fed on CDAA diet, determined a number of critical results in which KO mice showed: i) a marked decrease in steatosis in KO mice vs WT mice, as evident from the macroscopic aspect of livers (Figure 1A), hematoxylin/eosin staining (Figure 1B) and histopathological score (Figure 1C); ii) a decrease of transcript levels of markers of inflammatory response like IL-1β, TNF-α and the chemokine CCL-2 (Figure 1D), fully confirming results of the recently published study in which KO mice fed on CDAA diet were showing significantly less inflammatory response and infiltrate [13]; iii) a decrease in extracellular matrix deposition (i.e., liver fibrosis) as shown by Sirius Red staining (Figure 1E) and by related image analysis of liver sections (Figure 1F) as well as by a significant decrease in transcript levels for fibrogenesis markers like TGF-β1, α-SMA and collagen 1A1 (Figure 1G).

**Figure 2:**
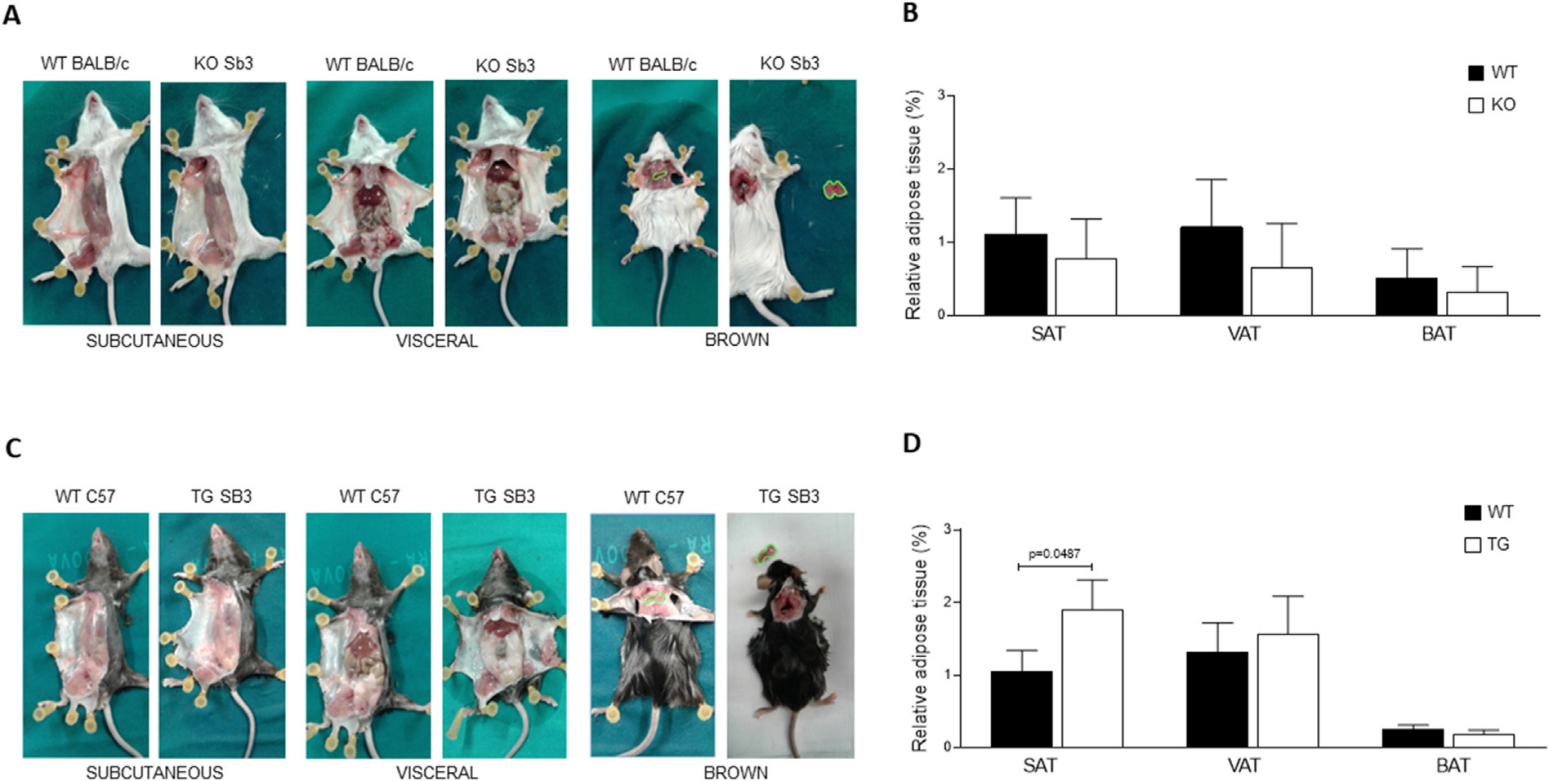
**Dysregulation of fat distribution in mice with different expression of SerpinB3.** A) Representative examples of subcutaneous or visceral white fat and of brown fat distribution are shown after skin removal in BALB/c mice lacking the Serpinb3a reactive site loop (KO) and in the corresponding BALB/c controls fed with normal diet. B) Values of relative adipose tissue in interscapular brown adipose tissue (BAT), subcutaneous adipose tissue (SAT) and in visceral adipose tissue (VAT), expressed as percentage of fat on total body weight (grams) in the WT and KO groups of mice. The results are reported as mean ± SEM (Unpaired t test with Welch's correction). C) Representative examples of subcutaneous or visceral white fat and of brown fat distribution in SerpinB3 transgenic (TG) mice and in the corresponding C57BL/6 controls fed with normal diet. D) Values of relative adipose tissue in interscapular brown adipose tissue (BAT), subcutaneous adipose tissue (SAT) and in visceral adipose tissue (VAT), expressed as percentage of fat on total body weight (grams) in the WT and TG groups of mice. The results are reported as mean ± SEM (Unpaired t test with Welch's correction). (For interpretation of the references to color in this figure legend, the reader is referred to the Web version of this article.)

These data overall indicate that KO mice are less prone to develop NASH than related WT mice, supporting the critical role of Serpinb3a in this experimental setting. Essentially similar results were obtained in the second experimental protocol of NASH used in this study, in which KO mice were fed on MCD diet. However, because this protocol is more aggressive and less translatable to human conditions of NASH, statistically significant differences were achieved only for some of the investigated parameters (Suppl. Figure 1 A-E), including the decrease in IL-1β and CCL-2 transcript levels (Suppl. Fig. 1B), the decrease in collagen deposition (Suppl. Fig. 1C, D), TGF-β1 transcript levels (Suppl. Fig. 1E). Moreover, at basal level, KO mice were characterized by a decreasing trend of adipose tissue deposition in subcutaneous -, visceral - and brown - adipose tissue (SAT, VAT and BAT, respectively) (Figure 2A,B). It should be noted that an opposite profile was observed in untreated TG mice (i.e., mice overexpressing SerpinB3 in hepatocytes), with this time a significant increase in SAT compartment (Figure 2C,D).

### 2.2 1-Piperidin Propionic acid protects from NASH development and progression

As a first step, we have checked the cytotoxicity of 1-PPA both *in vitro* and *in vivo*. For the first purpose, we employed the *in vitro* model of HepG2 cells stably transfected to overexpress SerpinB3, hereafter indicated as HepG2/SB3 cells, that we already used in previous studies [19,20]. Of interest, 1-PPA exerted significant cytotoxicity and then reduced viability of HepG2/SB3 cells only when used at much higher concentrations (>5 μg/ml) (Suppl. Fig. 2A). Moreover, 1-PPA significantly affected cell proliferation (followed for 70 h) at similarly higher concentrations, with a calculated EC50 value of 139.9 uM (Suppl. Fig. 2B). The compound 1-PPA was also injected *in vivo* in WT C57BL6/J mice (i.e. mice with the background of mice genetically manipulated to overexpress SerpinB3 in hepatocytes that we used to test 1-PPA *in vivo*) to test its cytotoxicity at doses of 70 and 700 ng/g b.w. The dose of 70 ng/g b.w, used as experimental treatment in the NASH animal model, did not affect significantly liver and kidney biochemical parameters and histological features, a part from a mild interstitial lymphocyte inflammation of the kidney, while the dose of 700 ng/g b.w. determined a significant increase of bilirubin and a trend toward increased values of creatinine (Suppl. Fig. 2C and Suppl. Table 1).

To test the effect of the small molecule 1-Piperidin Propionic acid (1-PPA), an inhibitor not only of SerpinB3 synthesis [28,29], but also of PAR2 [30], we have analyzed the real efficacy of the compound in inhibiting SerpinB3, C/EBPβ and PAR2 expression in the HA22T/VGH cell line which constitutively expresses SerpinB3. Very low concentrations of 1-PPA significantly down-regulated all these molecules at protein and transcription level in a dose-dependent manner up to 1 ng/ml concentration (Suppl. Fig. 3).

The *in vivo* efficacy of 1-PPA was tested on WT and transgenic mice on the same C57BL6/J background, but genetically manipulated to overexpress SerpinB3, hereafter indicated as TG/SB3 mice. WT and TG/SB3 mice were fed on CDAA diet for 12 weeks, as previously described [12,13], and treated or not with 1-PPA (70 ng/g b.w., i.p.) to investigate the effects of 1-PPA on inflammatory and fibrogenic responses.

**Figure 3:**
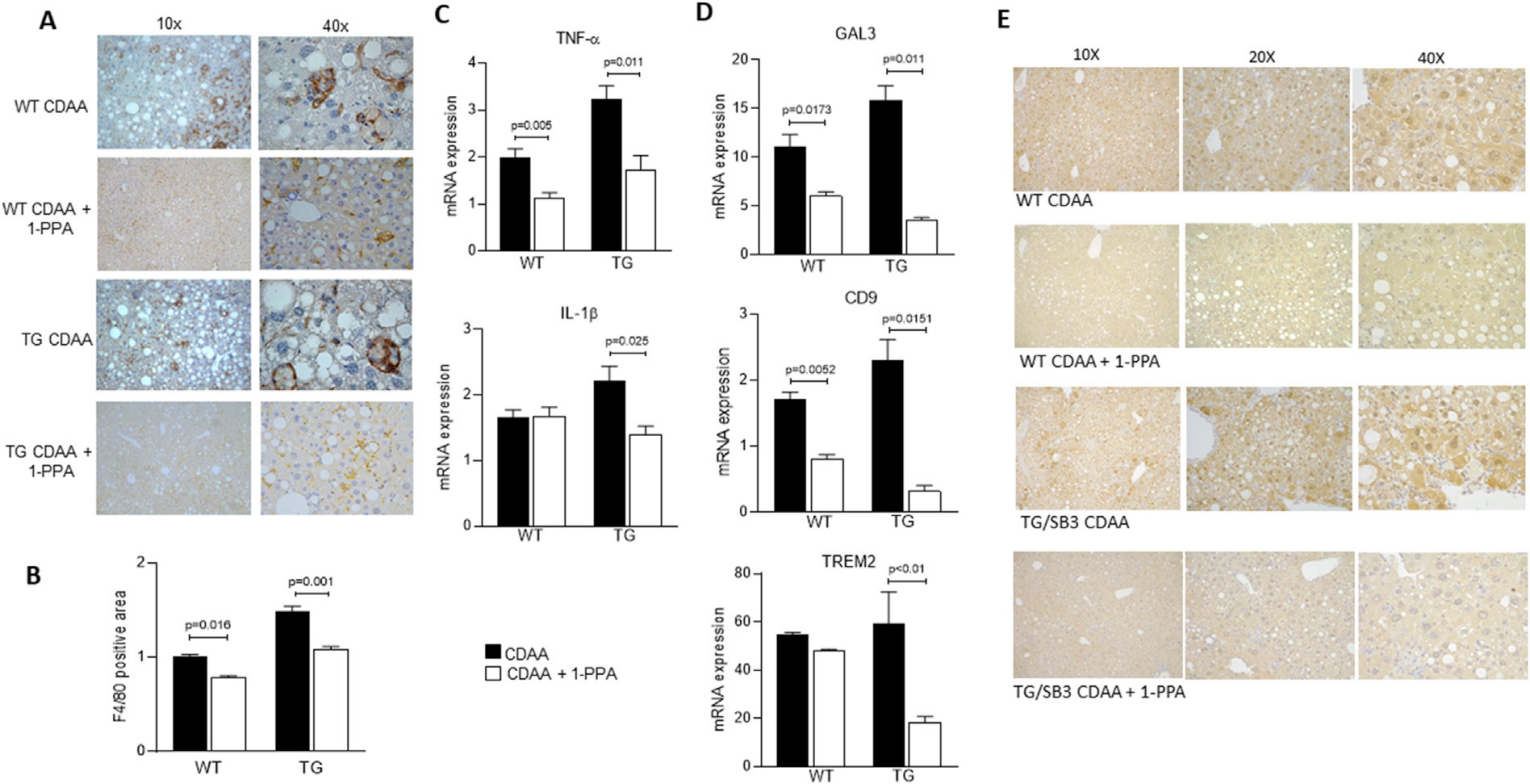
**Inhibitory activity of 1-Piperidin Propionic acid on inflammatory response in mice fed with CDAA diet.** A) Representative examples of macrophage infiltration, detected by F4/80 immunostaining, in the liver of control mice (WT) and of SerpinB3 transgenic mice (TG) injected or not with 1-PPA. B) Densitometric analysis of F4/80 immunostaining in the liver of the corresponding groups of mice (7 animals/group). C) mRNA levels of inflammatory genes, including TNF-α and IL-1β, in control mice (WT) or in SerpinB3 transgenic mice (TG), fed or not with CDAA diet and injected or not with 1-PPA. D) mRNA levels of markers of NAMs, Galectin 3 (GAL3), CD9 and TREM2, in control mice (WT) or in SerpinB3 transgenic mice (TG), fed or not with CDAA diet and injected or not with 1-PPA. E) Representative results of SerpinB3 immunostaining in the liver of control mice (WT) and of SerpinB3 transgenic mice (TG) fed with CDAA and injected or not with 1-PPA. Cytoplasmic and or nuclear brown color refers to SerpinB3 immune reactivity. The results are reported as mean ± SEM (Unpaired t test with Welch's correction). mRNA levels are expressed as 2^−ΔΔCT^. (For interpretation of the references to color in this figure legend, the reader is referred to the Web version of this article.)

Concerning inflammatory response, we confirmed that liver specimens from TG/SB3 mice fed on CDAA diet were characterized, when compared to specimens from WT mice, by a significant increase in inflammatory infiltrate of F4/80 positive macrophages (Figure 3A,B), which were often aggregated to form the typical so-called *“crown-like structures”* (Figure 3A), and an increase in IL-1β and TNF-α transcript levels (Figure 3C), as recently reported [13]. Of interest, all these parameters were significantly reduced in TG/SB3 mice (some also in WT mice) following *in vivo* 1-PPA administration (Figure 3A,C). In particular, we noted not only a significant reduction of F4/80 positive macrophage infiltration and of IL-1β and TNF-α transcript levels, but also a very evident disappearance of *“crown-like”* aggregates of macrophages (Figure 3A). Along these lines, since literature reports that NASH-associated macrophages (NAMs) are involved in forming crown-like structures in NASH livers and that SerpinB3 influences the hepatic levels of NAMs markers CD9, TREM2 and Galectin 3 (13), we have investigated whether 1-PPA was able to modulate the expression of these markers. Interestingly, 1-PPA significantly reduced the transcript levels of Galectin 3, CD9, and TREM2 in TG/SB3 mice fed on CDAA diet (Figure 3D). It is worth to note that SerpinB3 expression, that was detectable in WT mice fed with CDAA, although less evident than in TG/SB3 mice, was markedly reduced in mice treated with 1-PPA of both strains (Figure 3E).

Data obtained in mice fed on CDAA were essentially confirmed also in TG/SB3 mice fed on MCD diet: in these mice 1-PPA administration reduced infiltration of F4/80 positive macrophages (Suppl. Figs. 4A and B), “*crown-like*” aggregates of macrophages (Suppl. Fig. 4A) and IL-1β, TNF-α transcript levels (Suppl. Fig. 4C).

**Figure 4:**
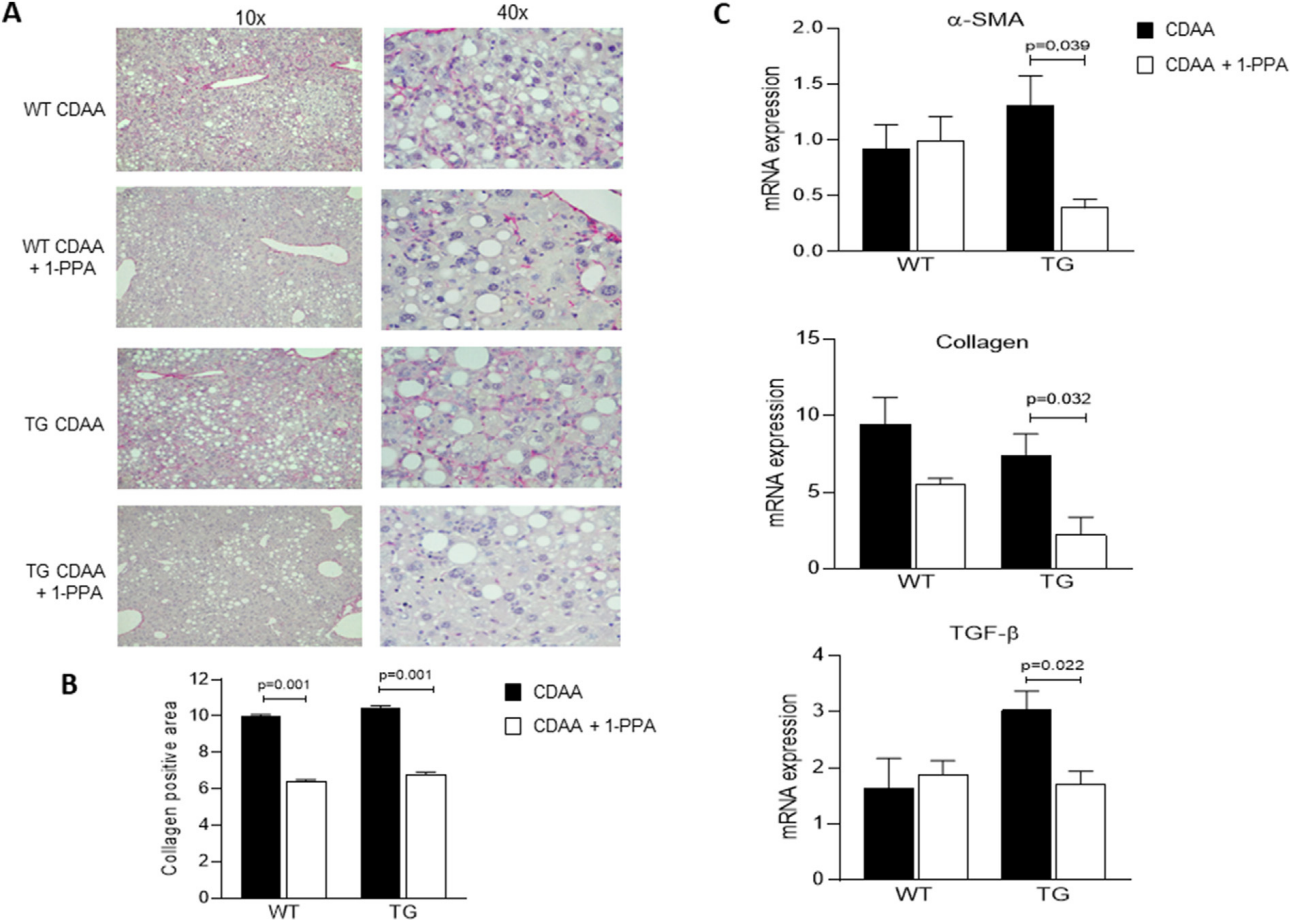
**Inhibitory activity of 1-Piperidin Propionic acid on fibrosis in mice fed with CDAA diet.** A) Representative examples of Sirius red staining in paraffin sections of mice livers in WT and in TG mice injected or not with 1-PPA. B) Densitometric analysis of collagen deposition measured after Sirius red staining in the liver of the corresponding groups of mice (7 animals/group). C) mRNA levels of fibrosis genes in the different groups of mice, including α-SMA, collagen 1A1 and TGF-β. The results are reported as mean ± SEM (Unpaired t test with Welch's correction). mRNA levels are expressed as 2^- ΔΔCT^. (For interpretation of the references to color in this figure legend, the reader is referred to the Web version of this article.)

Similar results were obtained by analyzing parameters related to fibrogenesis. Histochemical Sirius Red staining (Figure 4A), by confirming the increased deposition of extracellular matrix in TG/SB3 mice vs WT mice, showed that administration of 1-PPA significantly reduced fibrosis in both TG/SB3 and WT mice (Figure 4A), as confirmed by image analysis of collagen positive areas (Figure 4B). Similarly, in TG/SB3 mice 1-PPA administration resulted in a significant down-regulation of the transcript levels for α-SMA, collagen 1A1 and TGF-β1 (Figure 4C). Once again, homologous results were obtained when parameters related to fibrogenesis were investigated in TG/SB3 mice fed on MCD diet: 1-PPA administration reduced also in these mice the steatosis score (Suppl. Fig. 5A), Sirius Red staining and collagen positive area (Suppl. Fig. 5B) and transcript levels of critical parameters (Suppl. Fig. 5C).

**Figure 5:**
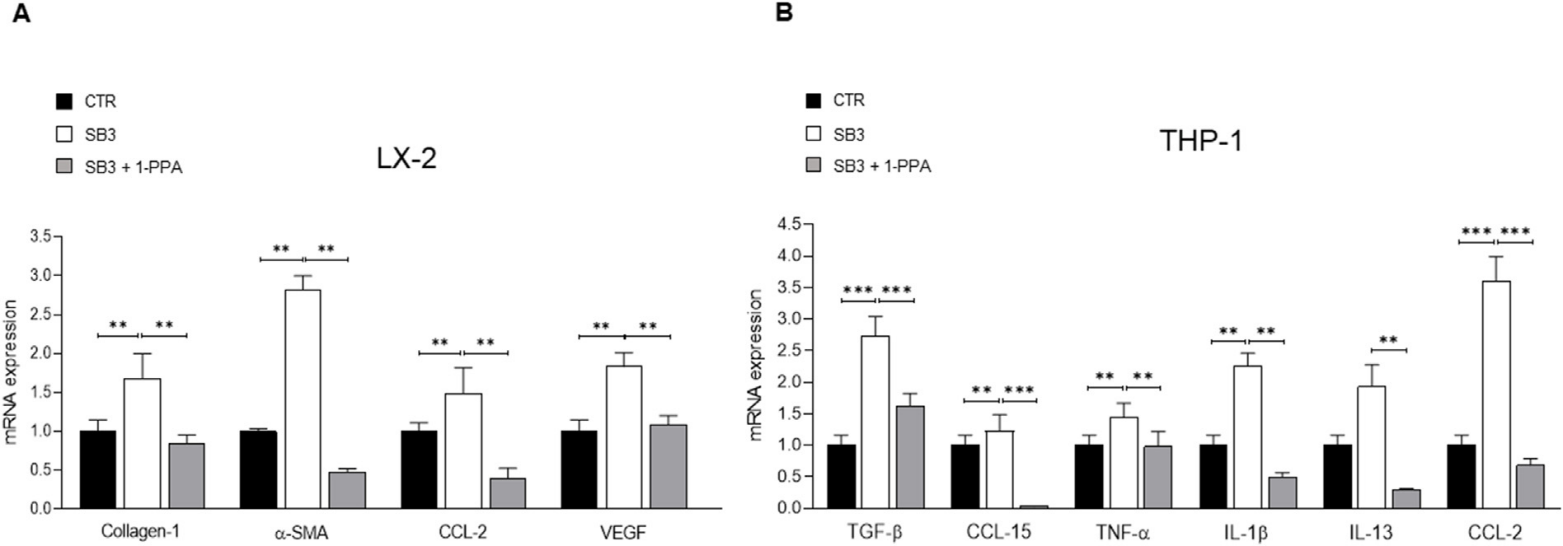
**Inhibitory effect of 1-Piperidin Propionic acid on fibrogenesis and inflammatory response *in vitro*.** A) Profile of fibrosis genes (collagen 1A1, α-SMA, CCL-2 and VEGF-A) in LX2 cells in presence of SerpinB3 (SB3, 200 ng/ml) alone or mixed with 1-PPA at 100 ng/ml concentration (SB3 + 1-PPA) after 24 h incubation. B) Profile of inflammatory genes (TGF-β, CCL-15, TNF-α, IL-1β, IL-13, CCL-2) in THP1 cells exposed to human recombinant SerpinB3 (SB3, 100 ng/ml) alone or mixed with 1-PPA at 100 ng/ml concentration (SB3 + 1-PPA) after 24 h incubation. The results are reported as mean + SEM of at least three experiments (Unpaired t test with Welch's correction). mRNA levels are expressed as 2^- ΔΔCT^.

Data on the effect of 1-PPA on inflammatory response and fibrogenesis were essentially confirmed when the compound was directly employed on either LX2 cells (a human immortalized myofibroblast cell line) or THP-1 cells (a cell line of human macrophages) exposed to human recombinant SB3 (hrSB3). The results obtained for LX2 cells indicate that 1-PPA was able to down-regulate in these cells hrSB3 – induced transcription for critical genes like collagen 1A1, α-SMA, CCL-2 and VEGF-A (Figure 5A). Similarly, 1-PPA down-regulated transcript levels of TNF-α, IL-1β, CCL-2, CCL-15, IL-13 and TGF-β1 that were induced by hrSB3 in THP-1 cells (Figure 5B).

### 2.3 PAR2 inhibition by 1-Piperidin Propionic acid prevents the SerpinB3 induced promoter CEBP-β up-regulation

To better understand the mechanistic behaviour of 1-PPA, the possibility of a direct interaction of 1-PPA with SerpinB3, beside PAR2 steric inhibition [30], was explored. For this purpose, two different biophysical approaches were tested. The thermal shift assay was used to evaluate whether the possible interaction with 1-PPA could modify the thermic stability of this serpin. As reported in Suppl. Fig. 6A, the denaturation curves showed irrelevant T melting differences, within the instrumental error (±2 °C), when SerpinB3 (5 μM) was mixed with different concentrations of 1-PPA, ranging from 5 to 500 μM.

To further confirm these data, the interaction was examined with isothermal titration calorimetry, widely used to assess biomolecular interactions, and also this approach provided negative results (Suppl. Fig. 6B). The potential difference (μW) was indeed evaluated and no binding model was identified, confirming that the compound does not interact directly with SerpinB3.

**Figure 6:**
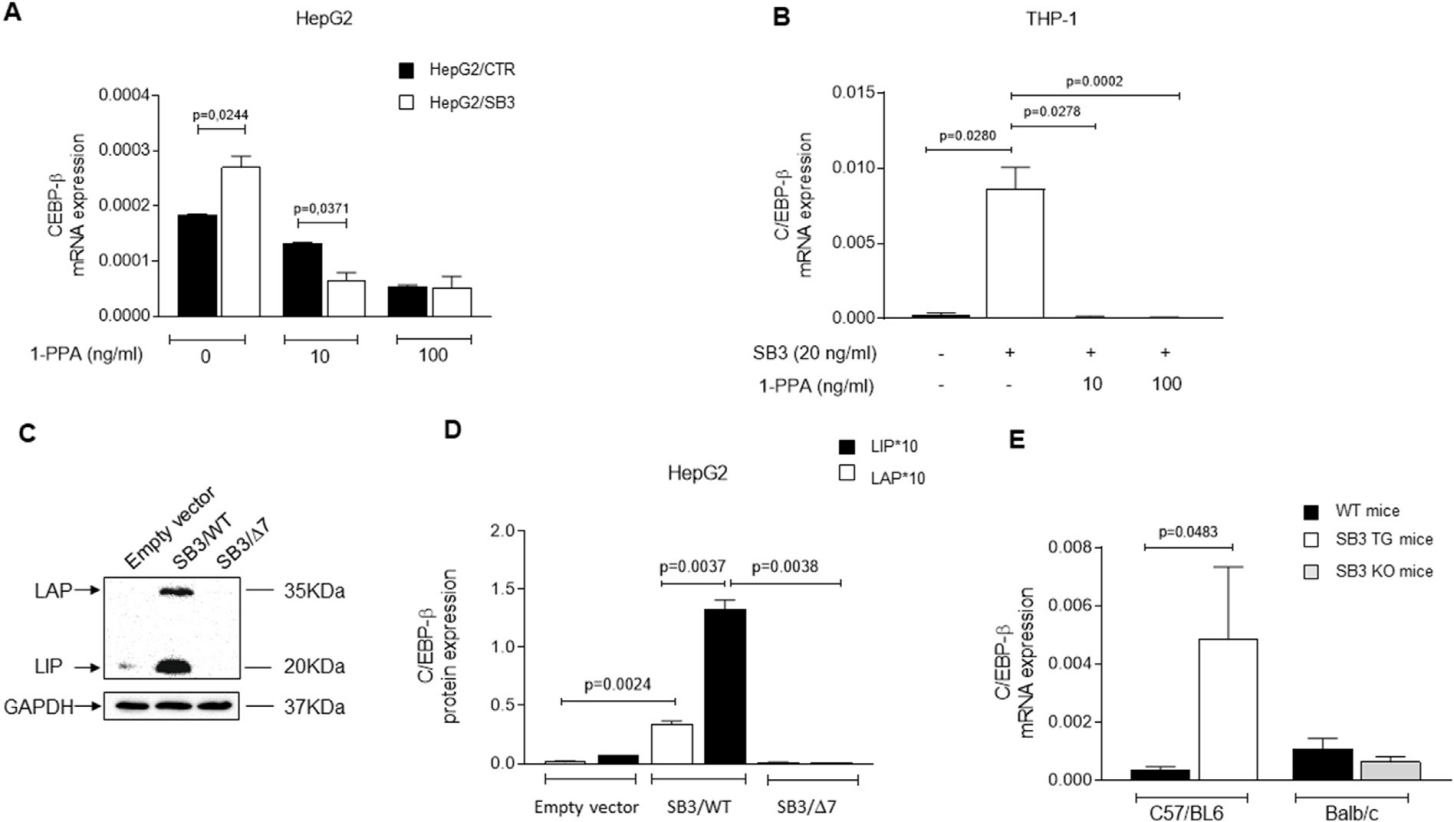
**1-Piperidin Propionic acid reduces CEBP-β transcription.** A) CEBP-β expression in HepG2 cells transfected with the plasmid vector alone (HepG2/CTR) and in HepG2 cells transfected to overexpress SerpinB3 (HepG2/SB3) in presence of increasing concentrations of 1-PPA or of Medium alone. B) CEBP-β expression profile in the THP-1 cell line. This transcription factor, not detectable in THP-1 cells, is markedly induced by SerpinB3 and the effect is efficiently reverted by the addition of 1-PPA. C) Example of Western blot of the isoforms Liver Activating Protein (LAP) and Liver Inhibitory Protein (LIP) of CEPB-β in control HepG2 (empty vector), in HepG2 cells overexpressing SerpinB3 wild type (SB3/WT) and in HepG2 cells overexpressing SerpinB3 lacking the antiprotease activity (SB3/Δ7). D) Densitometric analysis of protein expression of Liver Activating Protein (LAP) and of Liver Inhibitory Protein (LIP) of CEPB-β, normalized to GAPDH, in control HepG2 cells (Empty vector), in HepG2 cells overexpressing SerpinB3 wild type (SB3/WT) and in HepG2 cells lacking the antiprotease activity (SB3/Δ7). E) CEBP-β expression profile in C57BL6/J and BALB/c wild type mice (WT mice), SerpinB3 transgenic mice (SB3/TG) and mice deficient of the reactive site loop of Serpinb3a (SB3/KO).

On the basis of these results, and also considering the fact that 1-PPA determines transcriptional inhibition of SerpinB3 mRNA, we have assessed whether PAR2 activation could up-regulate possible promoters or enhancers for SerpinB3 gene, involved in metabolism and whether this effect could be abrogated following PAR2 inhibition by 1-PPA. Beside the already known up-regulator of SerpinB3 synthesis HIF-2α [19], previously found also to play a relevant role in lipid accumulation [21,22], in the human gene database GeneCards the CCAAT Enhancer Binding Protein Beta (CEPB-β) is listed. Since also this transcription factor is profoundly implicated in metabolic disturbances and in increased inflammatory response [33,35], we have assessed its expression in different cell lines with or without 1-PPA treatment and in mouse livers in relation to SerpinB3 expression. As expected, this transcription factor, that was found significantly up-regulated in HepG2 cells overexpressing SerpinB3, compared to control HepG2, was inhibited by 1-PPA in a dose dependent manner (Figure 6A). In the monocytic THP-1 cell line, the basal level the CEPB-β transcription factor was not detectable, but it was induced by exogenous hrSB3, suggesting a positive loop induction of both molecules. Also in this case, 1-PPA efficiently switched down CEPB-β expression, even at low concentration (Figure 6B). In addition, using HepG2 cell lines stably transfected to overexpress the wild type SerpinB3 form or the isoform lacking its anti-protease activity, obtained from HepG2 cells transfected with a mutant plasmid deleted in the SerpinB3 reactive site loop [7], we could demonstrate that the anti-protease activity of this serpin is essential for the protein induction of both the isoforms Liver Activating Protein (LAP) and Liver Inhibitory Protein (LIP) of CEPB-β [39] (Figure 6C,D). These data are in line with the results obtained in untreated mouse livers, where CEPB-β was significantly up-regulated in SB3/TG mice, compared to the corresponding wild type mice, while it was barely lower than in controls in SB3/KO mice (Figure 6E).

**Figure 7:**
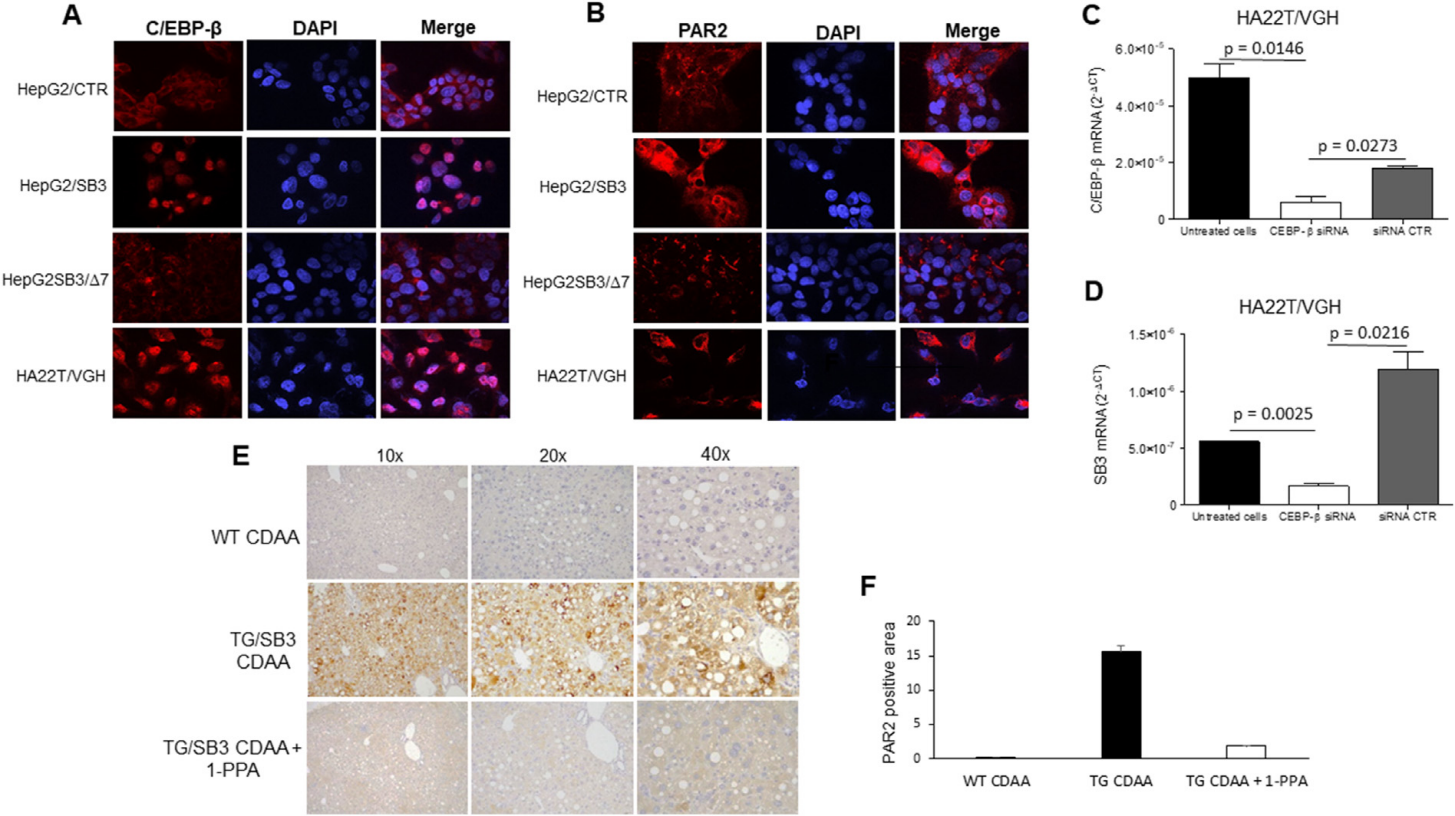
**C/EBP-β and PAR2 in relation to different extent of expression of SerpinB3.** Immunofluorescence results for C/EBP-β (Α) and for PAR2 (B) in HepG2 cells transfected with the plasmid vector alone (HepG2/CTR), in HepG2 cells transfected to overexpress SerpinB3 (HepG2/SB3), in HepG2 cells transfected with a plasmid vector carrying the SerpinB3 sequence deleted of 7aa in the reactive site loop (HepG2/SB3Δ7) and in the HA22T/VGH cells constitutively expressing SerpinB3. C) Effect of C/EBP-β silencing on the expression of C/EBP-β and of SerpinB3 (D) in the HA22T/HGV cell line. E) Representative results of immunostaining for PAR2 the liver in a wild type mouse (TG) and in SerpinB3 transgenic mice (TG/SB3) fed with CDAA and injected or not with 1-PPA. F) Densitometric analysis of PAR2 immunostaining in the liver of the corresponding groups of mice.

Along these lines, we analyzed *in vitro* cellular models carrying different expression of SerpinB3, namely, the HepG2 cell lines stably transfected to overexpress the wild type SerpinB3 form or the isoform lacking its anti-protease activity and the HA22T cell line that constitutively expresses SerpinB3 (Suppl. 3A). Immunofluorescence results not only confirmed the requirement of the integrity of the anti-protease activity of SerpinB3 to up-regulate and to determine nuclear translocation of CEPB-β (Figure 7Α), but also indicate that this is an essential requirement for the over-expression of PAR2. While HepG2/SB3 cells showed indeed a marked increase of this membrane receptor, compared to HepG2/WT, the lack of antiprotease activity carried by HepG2/Δ7 cells was associated to a reduced expression of PAR2 (Figure 7B). In addition, the HA22T/VGH cell line showed both up-regulation and nuclear translocation of CEPB-β and up-regulation of PAR2 (Figure 7A,B). In this cell line 1-PPA, beside the previously described ability to reduce SerpinB3 and of PAR2, determined also a significant reduction of CEPB-β transcription in a dose dependent manner (Suppl. Fig. 3C). On the other hand, silencing of CEPB-β led to a significant decrease not only of the target gene, but also of SerpinB3 (Figure 7C, D). The inhibitory effect 1-PPA on PAR2 was also confirmed in the liver of mouse fed with CDAA, where PAR2 was significantly up-regulated in SB3/TG mice, compared to the corresponding wild type mice and strongly reduced after 1-PPA treatment (Figure 7 E, F).

## 3 Discussion

While the successful treatments for hepatitis B and C have markedly reduced the burden of liver disease of viral etiology, non-alcoholic steatohepatitis (NASH) has emerged as a major cause of hepatic diseases in Western countries and it is expected to become the leading indication for liver transplantation and of liver-related mortality within a few years worldwide [40]. Despite the urgent need of effective therapies, approved drugs for NASH are still not available [41]. Several novel compounds, targeting different molecular events involved in the pathogenetic mechanism of NASH, including pathogenic metabolism, insulin resistance, inflammatory cell recruitment and pro-fibrogenic matrix profile, are currently in the pipeline, and while some of them failed in phase 2 or 3 clinical trials, others are providing promising results [5,42].

In the present study we provide evidence that SerpinB3-activated PAR2 can represent a novel molecular target for NASH. We have used the SerpinB3 and PAR2 inhibitor 1-PPA [[28], [29], [30]] to explore its ability to reduce the development of NASH in two different mouse models. PAR2 belongs to the protease-activated G-protein-coupled family of receptors and it has been recently described to play a pivotal role in metabolism control [31,32], beside to be a well-known driver of inflammatory responses [43]. Previous studies have reported that pharmacological blockade of PAR2, via pepducin, a short peptide derived from a GPCR intracellular loop, effectively suppressed NASH features in mice fed with MCD diet, although the mechanistic basis of the PAR2-promoting effect on liver steatosis in the MCD model was not yet known [44].

Literature reports that PAR2 is activated by trypsin-like proteases, such as tryptase released by mast cells during inflammation [44,45], matriptase [46] and, with a crosstalk between coagulation and inflammation, also by coagulation factors VIIa and Xa [47,48], when tissue factor expression levels are up-regulated as occurs in subjects with fibrotic liver disease. The interaction of PAR2 with these proteases during tissue injury and remodelling creates a microenvironment that can trigger a long-term pathological activation of PAR2 signalling pathway, including activation of mitogen-activated protein kinases involved in inflammation, proliferation, and differentiation of mesenchymal cells via IL-1β, TNF-α, TGF-β and NF-κB pathways [49].

The newly described essential role of the anti-protease activity of SerpinB3 was supported by the results obtained in genetically modified mice, since mice lacking the anti-protease activity of SerpinB3 not only had lower fat accumulation in basal conditions, but were also less prone to develop NASH, compared to control littermates. These findings were supported by lower fibrogenic and inflammatory responses following both CDAA and MCD diets. Conversely, transgenic mice overexpressing SerpinB3 developed a more intense liver damage, compared to the corresponding wild-type strain, characterized by increased fat accumulation, higher features of fibrosis and higher inflammatory response. These results corroborate previous findings reporting a pivotal role of SerpinB3 in the activation of the fibrogenic and inflammatory responses in NASH experimental models [12,13]. Further studies are required to identify the specific protease(s) involved in the interaction with SerpinB3, determining the activation of the PAR2 cellular receptor.

The small molecule 1-PPA, while did not induce significant cell and organ toxicity, was able to inhibit both PAR2 and SerpinB3 synthesis in a dose-dependent manner at very low concentrations. The inhibitory effect on SerpinB3 was associated to a parallel reduction in the synthesis of all the molecules induced by either the endogenous serpin or by its paracrine effect, as observed in the monocytic and stellate cell lines. The results obtained in experimental animals confirmed these findings and the weekly injection of 1-PPA was effective to markedly reduce the features of NASH not only in wild type mice but also in SerpinB3 overexpressing mice. In particular, as recently reported [13], SerpinB3 was able to influence the hepatic levels of Galectin 3, CD9 and TREM2, typical markers of NAMs, a population of hepatic macrophages emerging in progressive NAFLD-NASH and strictly associated with severity of steatosis, inflammation as well as fibrosis [50]. It is worth to note that 1-PPA was able to revert this effect by reducing hepatic levels of the typical markers of NAMs in mice transgenic for SerpinB3, suggesting a role of this compound in the shutdown of this class of macrophages induced by SerpinB3.

Concerning the mechanism of action of 1-PPA, it has been recently reported that it binds PAR2 in an allosteric pocket of the receptor inactive conformation, with antagonistic activity [30]. No information is available to date on its modality of SerpinB3 inhibition. To address this point, we have explored the possible direct interaction between 1-PPA and SerpinB3, but different biochemical approaches provided negative results. We have then explored the effect of this compound on C/EBP-β, a recently identified SerpinB3 transcription factors, documenting that it is upregulated in presence of SerpinB3, but only if carrying its active antiprotease activity. Several evidences indicate that C/EBP-β is profoundly involved in the processes related to metabolic syndrome [33] and these findings are in line with the fact that in our study mice lacking the active form of Serpinb3a presented at basal conditions a decreased fat mass and a trend toward lower levels of C/EBP-β. Like PAR2 [43] and SerpinB3 [51], C/EBP-β is activated in pro-inflammatory and ER stress conditions [52], and also these data are in agreement with the fact that our KO-mice presented a lower inflammatory response after CDAA or MCD diet, while opposite results were observed in SerpinB3 transgenic mice which already at basal level showed C/EBP-β levels higher than those of control mice. Concerning the involvement of this transcription factor in fibrogenesis, recent data indicate that alveolar accumulation of C/EBP-β in lung macrophages from patients and mice with pulmonary fibrosis causes upregulation of several profibrotic factors promoting pulmonary fibrosis, while its pharmacological degradation determines an effective therapeutic response against experimental fibrosis [37]. The compound 1-PPA was able not only to suppress PAR2 and SerpinB3, but also C/EBP-β transcription in hepatoma cells over-expressing SerpinB3.

On the basis of the results obtained, we propose that SerpinB3 is essential for PAR2 activation, which in turn up-regulates C/EBP-β, promoting SerpinB3 transcription. This cascade of events can be inhibited by 1-PPA that sterically determines PAR2 inhibition and blocks the described positive loop. Further studies are warranted in order to confirm whether the PAR2 inhibitor 1-PPA could be considered a clinically relevant strategy for NASH treatment and possibly, also for the modulation of liver cancer development, a growing risk in patients with non-alcoholic fatty liver disease, even in the absence of cirrhosis [53].

In conclusion, our study demonstrates that the PAR2 - C/EBP-β − SerpinB3 axis is relevant in the development of NASH. The small molecule 1-PPA is effective in the control of steatosis, inflammation and fibrosis in NASH experimental models through the inhibition of PAR2 activation and of its downstream molecules, like C/EBP-β and SerpinB3, implicated in metabolic alterations and NASH.

## 4 Materials and methods

### 4.1 Cell lines

Hepatoma cells (HepG2 cell line) (ATCC, Manassas, VA, USA), authenticated by BMR Genomics S.r.l. (Padova, Italy), according to PowerPlex® Fusion System protocol (Promega), have been engineered to stably overexpress SerpinB3, as previously described [54]. Briefly cells were transfected with a plasmid expression vector containing the whole human SerpinB3 gene, with a plasmid vector containing the SerpinB3 gene deleted of 7 aminoacids in the hinge region of the reactive site loop, lacking the antiprotease activity (Mutant B [7], kindly provided by Prof. Prof. TJ Harrison, University College, London) or with the plasmid vector alone (pcDNA3.1D/V5-His-TOPO™), as control, using Lipofectamine Reagent Plus (Invitrogen, Carlsbad, CA, USA). Transfected cells were selected using G418 (Geneticin) (Sigma–Aldrich, St. Louis, MO). Control HepG2 and HepG2 cells overexpressing whole SerpinB3 (HepG2/SB3) were incubated for 24 h with the SerpinB3 inhibitor 1-PPA (Sigma–Aldrich, St. Louis, Missouri, USA) at different concentrations (range 5–50 μg/ml) to assess cytotoxicity. For the assessment of 1-PPA cytotoxicity, real time cell proliferation was assessed using the xCELLigence instrument (ACEA Biosciences, Inc., San Diego, CA, USA), as previously described [55]. Dynamic cell proliferation was monitored at 15 min intervals and analyzed with RTCA software. The results were expressed as cell index value.

The human monocytic cell line THP-1 (kindly provided by Prof. Fabio Marra, University of Florence) and human LX2 cells (kindly provided by Prof. Scott L. Friedman, Icahn School of Medicine, Mount Sinai, New York) were cultured in Dulbecco's modified Eagle's medium (Sigma Aldrich Spa, Milan, Italy), supplemented with 10 % fetal calf serum and 1 % antibiotics. These cells, expressing trivial levels of SerpinB3, were incubated for 24 hrs with the recombinant entire SerpinB3 protein (hrSB3), obtained in our laboratory as previously described [56], at 200 ng/ml (LX2 cells) or at 100 ng/ml (THP-1 cells). Cells were simultaneously treated with 1-PPA at a concentration of 1 ng/mL to 100 ng/ml, or with medium supplemented with 10 % FCS alone and in the cellular extracts the transcriptional expression of different cytokines, including TGF-β, TNF-α, IL1-β, IL-13, CCL-2, CCL-15, VEGF, α−SMA, collagen 1A1, was analyzed.

The expression of SerpinB3, PAR2 and of the transcription factor CEBP-β was assessed in the cell lines described above and also in the hepatoma cells HA22T/VGH (kind gift of Prof. Giuseppina De Petro, University of Brescia) that express constitutively SerpinB3, in presence of 1-PPA at a concentration of 1 ng/ml to 100 ng/ml, or in medium supplemented with 10 % FBS, as control. For Western blot in cellular extracts, the following antibodies were used: mouse anti-SerpinB3 monoclonal antibody (1:200, Origene, Rockville, MD), rabbit anti-C/EBP-β monoclonal antibody (1:1000 dilution, ABCAM, Cambridge, UK), rabbit anti-PAR2 monoclonal antibody (1:1000 dilution, ABCAM, Cambridge, UK).

### 4.2 Molecular amplification techniques

In cell lines and in liver tissue samples total RNA was extracted using RNasy Trizol (Invitrogen, Carlsbad, CA, USA) according to the manufacturer's instructions. After determination of the purity and the integrity, total RNA, complementary DNA synthesis and quantitative real-time PCR reactions (qRT-PCR) were carried out as previously described [57] using the CFX96 real-time instrument (Bio-Rad Laboratories Inc, Hercules, CA, USA). Relative gene expression was normalized to the housekeeping genes and was calculated using the 2^−ΔΔCT^ method [58]. Specificity of the amplified PCR products was determined by melting curve analysis and confirmed by agarose gel electrophoresis. Human and mouse primer sequences used in the study are reported in Supplemental Table 2.

RNA interference experiments to knockdown C/EBP-β expression were carried out in HA22T/VGH cells by transient transfection. Cells (0.5 × 10^6^ cells/well) were seeded in 6 well plates containing or not cover glasses for immunofluorescence or molecular amplification analysis the day before. The siRNA for C/EBP-β and a negative siRNA control (Qiagen, Hlden, Germany) were transfected using Lipofectamine 3000 (Invitrogen-Waltham, Massachusetts, USA), following manufacturer's instructions. Cells were harvested 48 hrs post-transfection and glasses were fixed using 4 % PFA. The knockdown gene efficiency was confirmed by qRT-PCR and immunofluorescence analysis.

### 4.3 Animal models

#### 4.3.1 NASH experimental models

The study was carried out on C57BL/6 mice (12 weeks old) transgenic for human SerpinB3, (kindly provided by Prof. Cassani, Tecnogen, Caserta, Italy) [59], and their corresponding wild type C57BL/6 mice of similar age. BALB/c mice, deficient of the reactive site loop of Serpinb3a (Serpinb3-KO, originally kindly provided by Dr. Gary Silverman and Dr. Cliff J. Luke, Dept of Pediatrics, Washington University School of Medicine, St. Louis, MO) [38] have been also used and compared with wild type BALB/c mice of similar age as control.

All animals were kept under specific pathogen-free conditions and maintained with free access to pellet food and water at the Animal Care Facility of the Experimental Surgery Division of the University of Padua.

In order to induce experimental NASH, SerpinB3-transgenic (TG) and Serpinb3-KO (Sb3–KO) mice and their control littermates were fed on (a) the choline-deficient, l-amino acid defined (CDAA) diet (Laboratorio Dottori Piccioni, Gessate, Italy) for 12 weeks or (b) the methionine-choline deficient (MCD) diet (Laboratorio Dottori Piccioni, Gessate, Italy) for 8 weeks (7 animals/group). Parallel groups of TG and control mice, fed on CDAA diet (5 animals/group) or on MCD diet (5 animals/group), were injected weekly with 1-PPA (70 ng/g) or with the vehicle alone, starting from the third and second month and were sacrificed at week 12 and week 8, respectively. Liver samples were obtained, and one part was formalin-fixed and paraffin-embedded, while one part was immediately frozen at −80 °C for morphological or molecular analyses. Experiments and protocols were approved by the Animal Welfare Committee of University of Padua and by the Animal Investigation Committee of the Italian Ministry of Health (n° 442/2018-PR and n° 116/92), in accordance with European Union regulations.

#### 4.3.2 Fat assessment in different mice strains

Fat distribution was assessed in untreated SerpinB3 transgenic or knockout mice and in their corresponding wild type strains (5 animals/group). For the adipose tissue sampling, the mice were weighed and euthanized at the end of the experiment. The adipose tissues were dissected and weighed after death. In detail, dorsal and ventral external surfaces of the mouse were sterilized with 70 % ethanol to minimize contamination during dissection. The skin on the back was removed and a layer of white adipose tissue (WAT) was visible at the level of the shoulder blades. This layer covered the two lobes of interscapular brown adipose tissue (BAT). These two kinds of adipose tissue were dissected together and further separated outside the body, as the two tissue were clearly distinguishable based on their color. Similarly, after removal of the skin at the level of the flanks of the body, there was a layer of subcutaneous adipose tissue (SAT) positioned against the rib cage on both sides that was also removed. Lining the intestines and attached to each epididymis and testis were the large vessel-rich adipose depots, the visceral adipose tissue (VAT) that was collected after its separation from epididymis and vas deferens.

### 4.4 Histochemistry, immunohistochemistry and Sirius Red staining

In paraffin-embedded murine liver sections, steatosis score was semi-quantified, after hematoxylin-eosin staining, using a four-tier scoring scale based on the extent of fat content (0 = negative; 1+ = < 10 %; 2+ = >10, ≥30 %; 3+ = >30 %). Immunohistochemistry for the monocytic marker F4/80, SerpinB3 and PAR2 was assessed as previously described [13]. Collagen deposition was analyzed by Picro-Sirius Red staining. Quantification of immunostaining and fibrosis were assessed by histomorphometric analysis and images were then analyzed using ImageJ software (National Institutes of Health, Bethesda, MD), as previously described [13].

### 4.5 Immunofluorescence

HepG2 and HAT22/VGH were seeded on slides (4 × 10^5^ cells/slide) and cells were fixed with 4 % paraformaldehyde, permeabilized with 0.4 % Tryton X-100, and blocked with 5 % goat serum (Invitrogen Life Technologies, Waltham, MA, USA) in PBS containing 1 % BSA. Slides were incubated with rabbit monoclonal anti-C/EBP-β and anti-PAR2 (Abcam-Cambridge UK) antibodies for 1 h at room temperature, followed by incubation with the anti-rabbit Alexa Fluor 546 (Invitrogen Life Technologies, Waltham, MA, USA) secondary antibody. Cellular nuclei were counterstained with Dapi (Merck KGaA, Darmstadt, Germany). Slides were mounted with ELVANOL (Merck KGaA, Darmstadt, Germany) and observed under a fluorescence microscope (Axiovert 200M-Apotome.2, Carl Zeiss MicroImaging GmbH, Göttingen, Germany).

### 4.6 1-Piperidin Propionic acid animal toxicity

To assess 1-PPA toxicity, 6 weeks old C57BL/6 mice, kept with free access to pellet food and water, were injected weekly with the 1-PPA concentration used in the NASH models (70 ng/g) or with 10 times higher 1-PPA concentration (700 ng/g) for 26 weeks (3 mice/group), a time length that is two to three-fold higher than that of the NASH models. Blood samples were achieved by tail vein puncture before starting 1-PPA injections, while at the end of the study animals were sacrificed and blood samples were taken by cardiac puncture. Kidney and liver samples were removed, formalin fixed and paraffin embedded for histologic examination. In collected serum samples alanine amino transferase (ALT) and bilirubin were analyzed to assess liver damage, while serum creatinine was analyzed to assess kidney function.

### 4.7 Biophysical analysis of 1-piperidin propionic acid-SerpinB3 interaction

To verify whether 1-PPA was physically interacting with SerpinB3, as possible mechanism of action, the following approaches were used.

#### 4.7.1 Differential scanning fluorimetry

Purified recombinant human SerpinB3, obtained in our laboratory, as previously described [56], was incubated at 5 μM concentration with increasing concentration of 1-PPA (0, 5, 50, 500 μM) and SYPRO® ORANGE (Thermofisher Scientific) 8x. The samples were prepared in triple technical replicates and solubilized in PBS pH 7.4. The samples were then thermically denatured, with a linear gradient of +0.5 °C/min, and the fluorescence was measured with StepOne® Real-Time PCR System (Thermofisher Scientific).

#### 4.7.2 Isothermal titration calorimetry

SerpinB3 was buffer exchanged in a 50 mM MES pH 5.5, 500 mM NaCl with final concentration of 57 μM and 1-PPA was solubilized in the same buffer at 10 mM concentration. Isothermal titration calorimetry (ITC) experiments were carried out in PEAQ-ITC Calorimeter (Malvern) at 25 °C. The analysis was performed with recombinant SerpinB3 at 57 μM and 1-PPA was used as titrant at 2 mM in the injection syringe. The measure was carried for 40 injections and while the sample was stirred. Data were analyzed with Microcal PEAQ-ITC software.

### 4.8 Statistical analysis

Statistical significance was determined by non-parametric procedures using the Student's t test or ANOVA for analysis of variance. Normality of distribution for quantitative variables was assessed by Kolmogorov and Smirnov test. The calculations were carried out with Graph Pad InStat Software (San Diego, CA, USA). The null hypothesis was rejected at P < 0.05. Data in bar graphs represent means ± SEM and were obtained from at least three independent experiments. Western-blot and morphological images are representative of at least three experiments with similar results.

## Author contributions

Conceptualization, V.G., N.E., P.M., V.R., P.P.; Methodology, T.C., S.Q., R.M., F.P., B.A., C.M., T.E., G.M., D.S.S., C.S., Formal analysis, V.G., N.E., P.M., V.R., P.F., G.M., C.L., D.S.S; Funding acquisition, P.M., V.R., P.P.; Investigation, V.M., N.E., T.C., M.A., P.F, G.M.; Project administration, P.P., P.M., R.V.; Resources, P.P., P.M., V.R.; Supervision P.P., P.M., R.V.; Validation, P.P., P.M.,V.R., A.M, C.L.; Visualization, V.G., T.C., P.P.; Writing original draft, V.G., T.C., M.A., N.E,; Writing review & editing, P.P., P.M., V.R.

## Declaration of competing interest

The authors declare the following financial interests/personal relationships which may be considered as potential competing interests: The authors declare that P.P., B.A., M.A., Q.S., R.M., T.C. and V.G. are listed as inventors of patent N. IT 102017000026858, European patent EP 392351 and P.P., B.A., L.C., M.C., Q.S., R.M., T.C. and V.G of the Italian Patent Application N. 102022000014593 filed by the University of Padova, PTC pending. No conflict of interest exists for the other authors.

## Data Availability

Data will be made available on request.
