# Supplementary material for: The protease activated receptor 2 - CCAAT/enhancer-binding protein beta - SerpinB3 axis inhibition as a novel strategy for the treatment of non-alcoholic steatohepatitis
Source: Mol Metab. 2024 Feb 1;81:101889. doi: 10.1016/j.molmet.2024.101889 (PMC10864841; doi:10.1016/j.molmet.2024.101889)
Supplement: Multimedia component 1 [file mmc1.pdf]

The Protease Activated Receptor 2 - CCAAT/Enhancer-Binding Protein beta - SerpinB3 axis inhibition as a novel strategy for the treatment of Non-Alcoholic Steatohepatitis

Villano Gianmarco[1*], Novo Erica[2*], Turato Cristian[3], Quarta Santina[4], Ruvoletto Mariagrazia[4], Biasiolo Alessandra[4], Protopapa Francesca[2], Chinellato Monica[4], Martini Andrea[4], Trevellin Elisabetta[4], Granzotto Marnie[4], Cannito Stefania[2], Cendron Laura[5], De Siervi Silvia[3], Guido Maria[4], Parola Maurizio[2**], Roberto Vettor[4**], Pontisso Patrizia[4**§].

[1]Dept. of Surgical, Oncological and Gastroenterological Sciences, University of Padova, Italy; [2]Dept. of Clinical and Biological Sciences, University of Torino, Italy; [3]Dept. of Molecular Medicine, University of Pavia, Italy; [4]Dept. of Medicine, University of Padova, Italy; [5]Dept. of Biology, University of Padova, Italy.

* Shared first authorship

** Shared senior authorship

SUPPLEMENTARY MATERIAL

SUPPLEMENTARY FIGURES

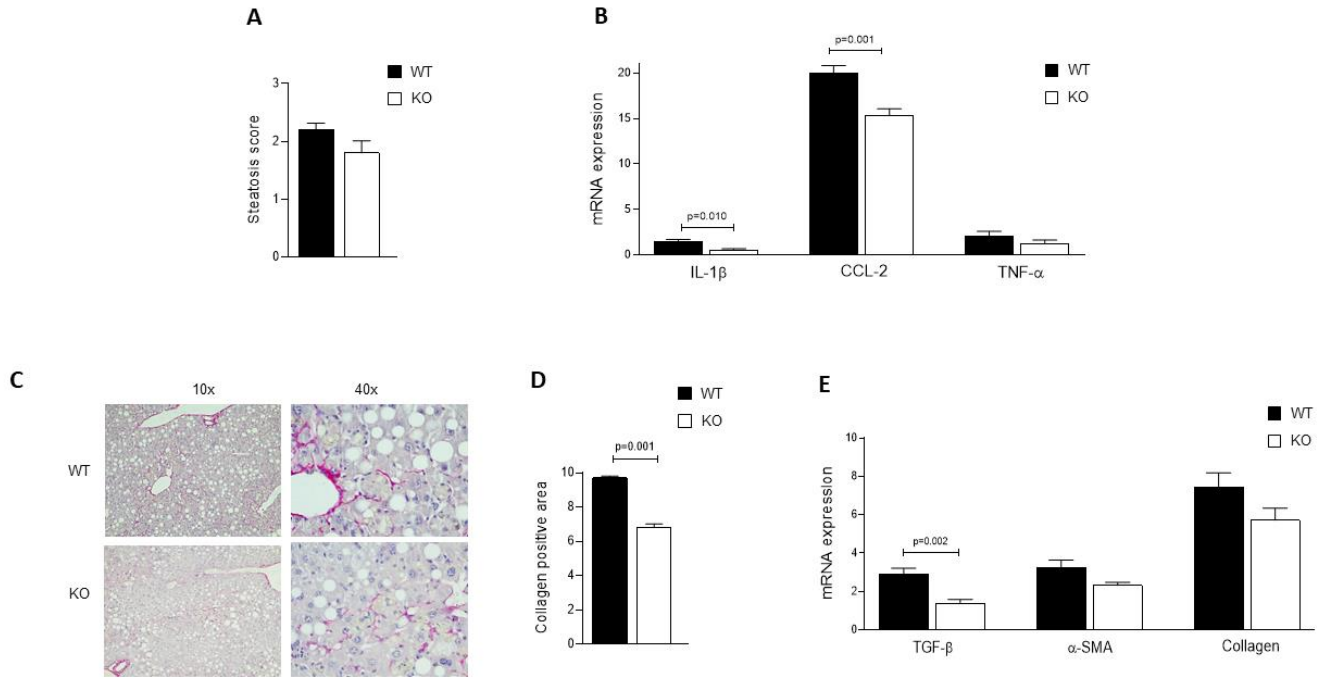

Supplementary Figure 1. Morphological and transcriptional findings in the liver after MCD diet in KO and control BALB/c mice. (A): Extent of steatosis score in KO mouse and in the BALB/c control mice. (B): Expression of genes involved in inflammation: IL-1β, CCL-2 and TNF-α. (C): Examples of Sirius red staining of the liver of a BALB/c wild type mouse (WT) and of a mouse deficient of the reactive site loop of Serpinb3a (KO), fed with MCD diet. (D): Densitometric analysis of collagen deposition measured after Sirius red staining in WT and KO mice. (E): Expression of genes involved in fibrosis: TGF–β, α-SMA and collagen 1A1. Columns represent mean values and bars refer to SEM. Levels of RNA gene expression are reported as $2^{-\Delta\Delta ct}$. N= 7 mice/group.

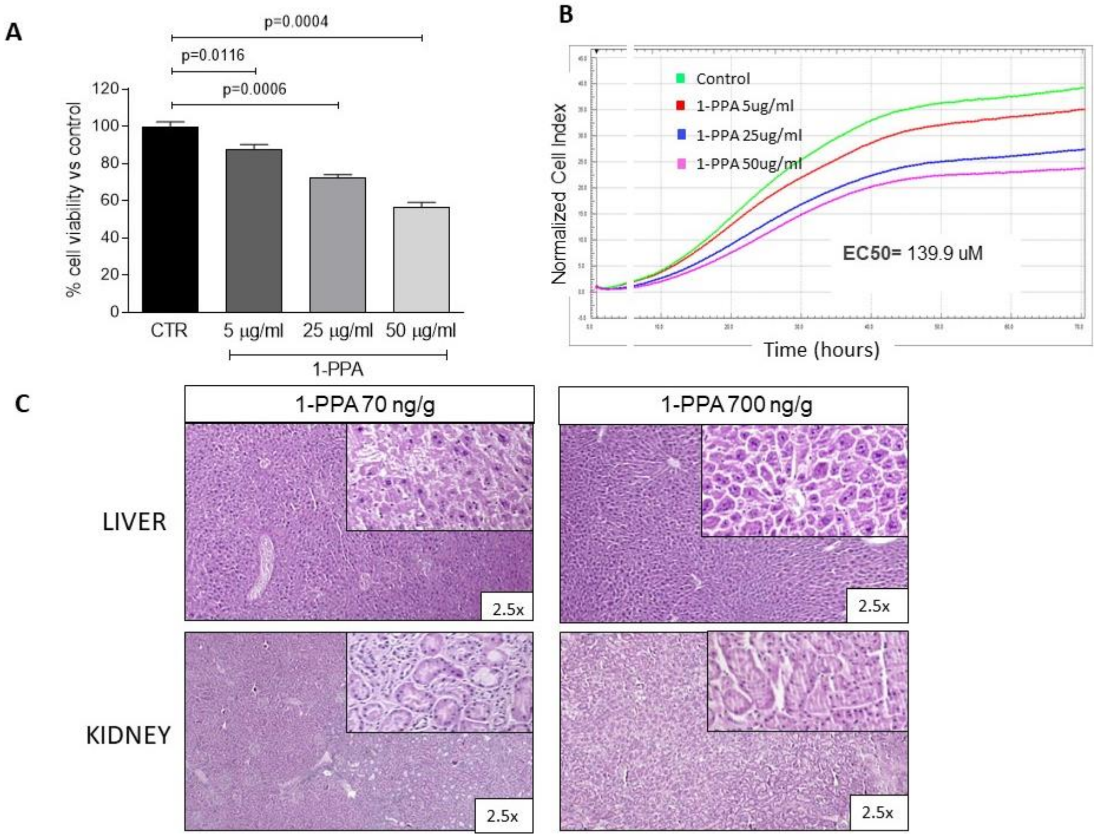

Supplementary Figure 2. Toxicity of 1-Piperidin Propionic acid. A) Cell viability calculated at 36 hours in the HepG2 cell line in control Medium or in presence of increasing concentrations of 1-PPA. Bars represent the mean values ± SEM of the results (Unpaired t test with Welch's correction) obtained in 6 wells for each experimental condition. B) Real time cell proliferation monitored over 70 hours in the HepG2 cell line in presence of increasing concentrations of 1-PPA. The results are expressed as normalized cell index. The EC50 has been calculated by the software of the XCelligence Instrument. C) Examples of histological features of the liver and of the kidney of mice intraperitoneally injected weekly with 1-PPA at the dose of 70 or of 700 ng/g for 26 weeks. Inserts magnification 10X.

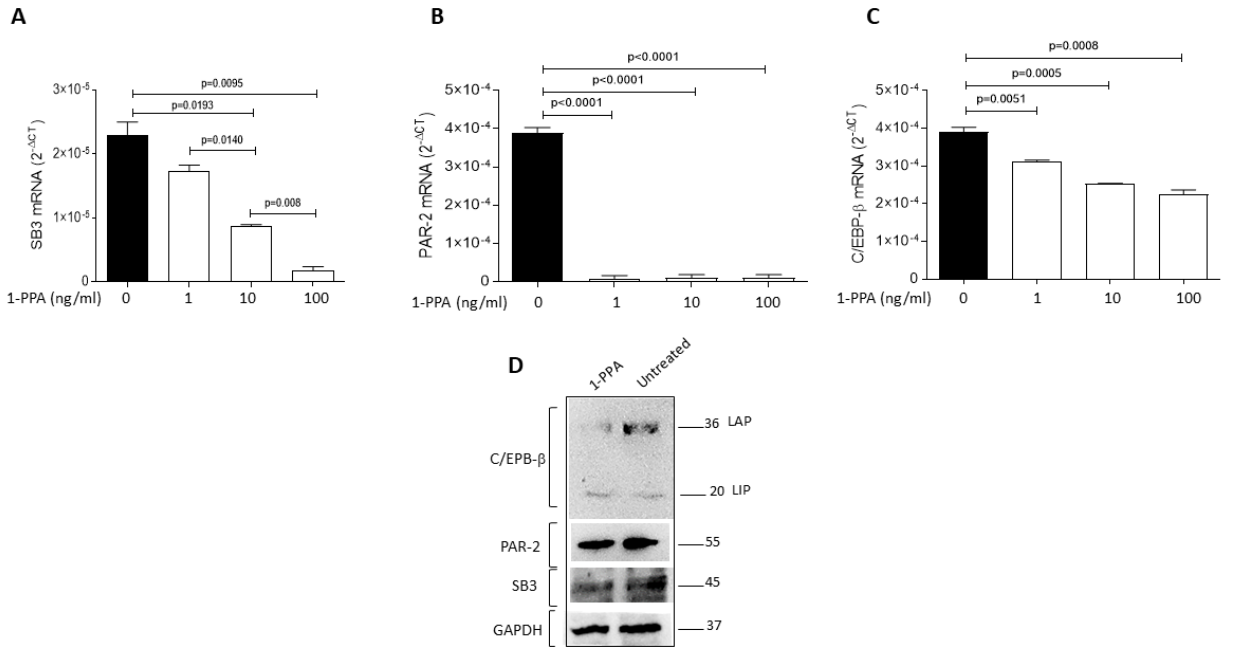

Supplementary Figure 3. Inhibitory activity of 1-Piperidin Propionic acid. Transcriptional levels of SerpinB3 mRNA (A), of PAR2 mRNA (B) and of C/EBP-β mRNA in HA22T/VGH cells obtained after 48 hours from seeding in medium, used as control (0) or in presence of different concentrations of 1-PPA. Levels of mRNA gene expression are reported as $2^{-\Delta\Delta CT}$. D) Example of C/EBP-β, PAR2 and SerpinB3 (SB3) detected by Western blot in HA22T/VGH cells after 48 hours from seeding in absence or in presence of 1-PPA (100 ng/ml).

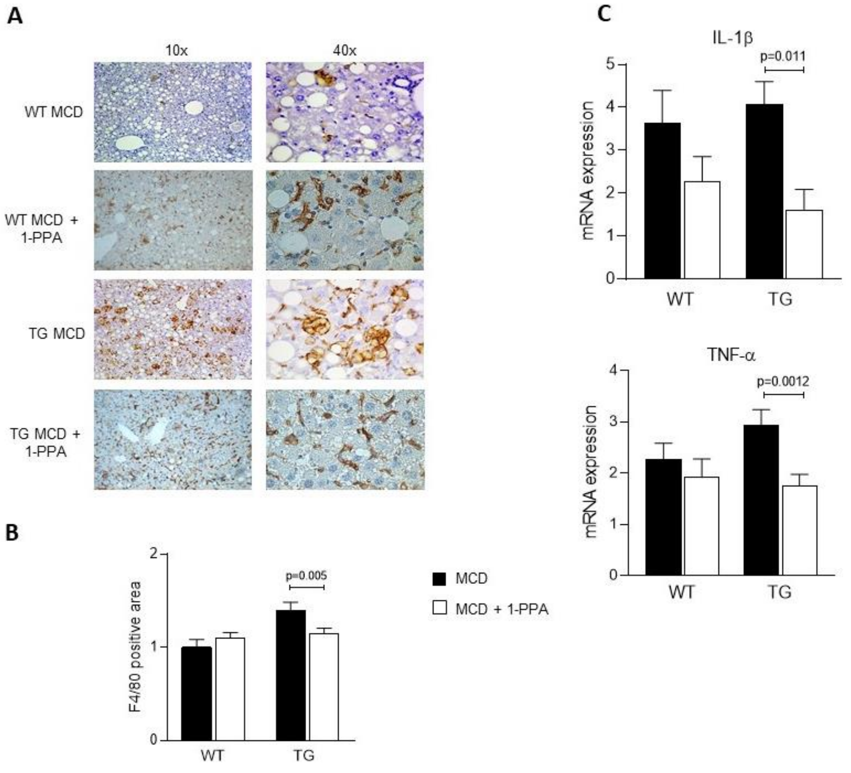

Supplementary Figure 4. Inhibitory activity of 1-Piperidin Propionic acid on inflammatory response in mice fed with MCD diet. A) Representative examples of macrophage infiltration, detected by F4/80 immunostaining, in the liver of control mice (WT) and of SerpinB3 transgenic mice (TG) injected or not with 1-PPA. B) Densitometric analysis of F4/80 immunostaining in the liver of the corresponding groups of mice (7 animals/group). C) RNA levels of inflammatory genes in the different groups of mice, including IL-1β and TNF-α. The results are reported as mean $\pm$ SEM (Unpaired t test with Welch's correction). RNA levels are expressed as $2^{-\Delta\Delta CT}$.

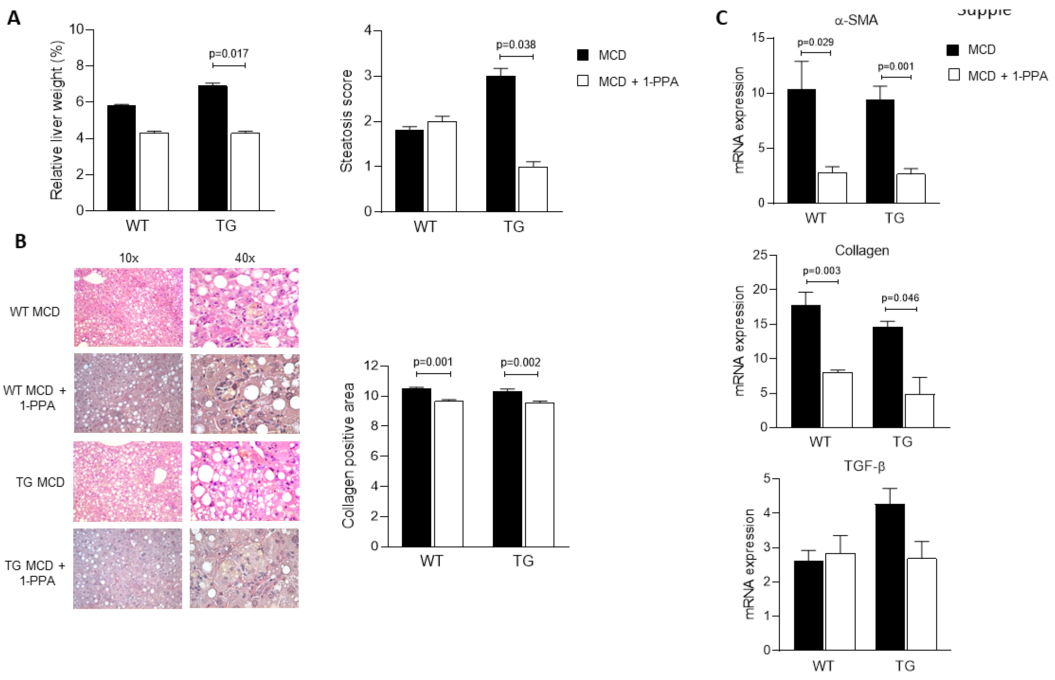

Supplementary Figure 5. Inhibitory activity of 1-Piperidin Propionic acid on steatosis and fibrosis in mice fed with MCD diet. A) Relative liver weight obtained at sacrifice after MCD diet and steatosis score in control mice (WT) and in SerpinB3 transgenic mice (TG) injected or not with 1-PPA (7 animals/group). B) Representative examples of Sirius red staining in paraffin sections of livers in WT and in TG mice fed with MCD and injected or not with 1-PPA. The right panel represents the densitometric analysis of collagen deposition measured after Sirius red staining in the corresponding groups of mice. C) RNA levels of fibrosis genes in the different groups of mice, including α-SMA, collagen 1A1 and TGF-β. The results are reported as mean $\pm$ SEM (Unpaired t test with Welch's correction). RNA levels are expressed as $2^{-\Delta\Delta CT}$.

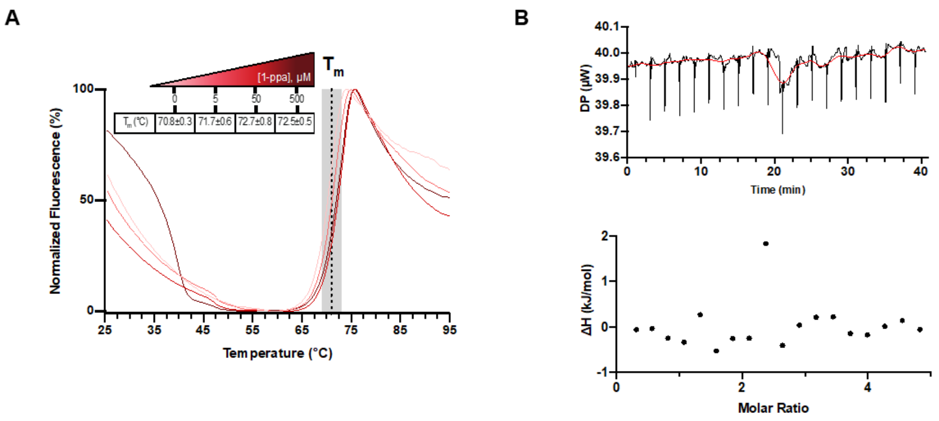

Supplementary Figure 6. Biophysical studies of interaction between SerpinB3 and 1-Piperidin Propionic acid. A) Differential Scanning Fluorimetry curves are reported as Relative fluorescence, normalized in percentage on the highest and lowest values of each dataset. The Melting Temperature (T_m) was calculated based on 3 technical replicates of each sample and it is shown with each Standard Deviation. B) Upper panel: Isothermal Titration Calorimetry assay, plot of heat changes in relation to molar ratio; Bottom panel represents the heat effects associated with 1-PPA injection. Both the techniques reveal that there is not interaction between SerpinB3 and 1-PPA.

SUPPLEMENTARY TABLES

Supplementary Table 1. Biochemical parameters of liver and kidney function in C57BL/6 mice injected weekly with the 1-PPA concentration of 70 ng/g or of 700 ng/g body weight (3 animals/group). All the parameters were assessed before 1-PPA injection (T_0) and after 26 weeks of weekly 1-PPA injection (T_{26}).

**C57BL/6J mice**	**1-PPA 70 ng/g**			**1-PPA 700 ng/g**		
	**T_0**	**T_{26}**	**p***	**T_0**	**T_{26}**	**p***
**ALT (U/L)**						
Median<br>Range	38.3<br>30-45	46.5<br>30-57	ns	39<br>36-42	46.5<br>39-54	ns
**Bilirubin (µmol/L)**						
Median<br>Range	0.53<br>0.1-0.9	0.75 $\pm$ 0.4<br>0-1.5	ns	0.45<br>0.1-0.9	1.65<br>1.2-2.1	0.0265
**Creatinine (µmol/L)**						
Median<br>Range	19.5<br>12-33	22.5<br>9-36	ns	34.5<br>24-45	55.5<br>42-69	ns

ALT: Alanine Amino Transferase. *Mann-Whitney test

Supplementary Table 2. Mouse and human primer sequences used in the study.

GENE	Forward primer	Reverse primer
**m-TGFβ**	5’-TTGCTTCAGCTCCACAGAGA-3’	5’-TGGTTGTAGAGGGCAAGGAC-3’
**m-α-SMA**	5'-GACGTACAACTGGTATTGTG-3'	5'- TCAGGATCTTCATGAGGTAG -3'
**m-Collagen 1**	5’-AAATCTGCACACTGCCAT GA-3’	5’-GCATGTTCGAAATCCAGTGA-3’
**m-IL1β**	5’-GAAATGCCACCTTTTGACAGTGAT-3’	5’-TTGGAAGCAGCCCTTCATCTT-3’
**m-CCL2**	5’-GCCTGCTGTTCACAGTTGC-3’	5’GAGTGGGGCGTTAACTGCAT-3’
**m-TNFα**	5’-AGCCCCCAGTCTGTATCCTT-3’	5’- CTCCCTTTGCAG AACTCAGG-3’
**m-CD9**	5’- TTCGCCATTGAGATAGCCGC-3’	5’-GCTATGCCACAGCAGTCCAA-3’
**m-TREM2**	5’-CCTGCAGAAAGTACTGGTGGA-3’	5’-TCTCTTGATTCCTGGAGGTGC-3’
**m-Gal3**	5’-CCACTTTAACCCCCGCTTCA-3’	5’-CAAAGGGGAAGGCTGACTGT-3’
**h-PAR2**	5’- GCTAGCAGCCTCTCTCTCCT-3’	5’- GTGGGATGTGCCATCAACCT-3’
**h-CEBP-β**	5'-AAGCACAGCGACGAGTACAA-3'	5'-ACAGCTGCTCCACCTTCTTC-3'
**h-Collagen 1**	5'-GTGCTAAAGGTGCCAATGGT-3'	5'-ACCAGGTTCACCGCTGTTAC-3'
**h-α-SMA**	5'-ACCCACAATGTCCCCATCTA-3'	5'-GAAGGAATAGCCACGCTCAG-3'
**h-CCL2**	5'-CCCCAGTCACCTGCTGTTAT -3'	5'-AGATCTCCTTGGCCACAATG-3'
**h-TGFβ**	5′-AAGTGGACATCAACGGGTTC-3′	5′-GTCCTTGCGGAAGTCAATGT-3′
**h-TNFα**	5'-AACCTCCTCTCTGCCATCAA-3'	5'-GGAAGACCCCTCCCAGATAG-3'
**h-IL1β**	5'-TGAAAGCTCTCCACCTCCAG-3'	5'-CACGCAGGACAGGTACAGAT-3'
**h-IL-13**	5'-GTACTGTGCAGCCCTGGAAT-3'	5'-TTTACAAACTGGGCCACCTC-3'
**h-VEGF**	5’ CCCACTGAGGAGTCCAACAT 3’	5’ TTTCTTGCGCTTTCGTTTTT 3’
**h-CCL 15**	5’-TCATGCTTGTTGCTGTCCTT -3’	5’-CACGGGATGCTTTGTGAGAT-3’

m, mouse; h, human
